# Incidence and risk factors for recurrent Henoch-Schönlein purpura in children from a 16-year nationwide database

**DOI:** 10.1186/s12969-018-0247-8

**Published:** 2018-04-16

**Authors:** Wei-Te Lei, Po-Li Tsai, Szu-Hung Chu, Yu-Hsuan Kao, Chien-Yu Lin, Li-Ching Fang, Shyh-Dar Shyur, Yu-Wen Lin, Shu-I Wu

**Affiliations:** 1 Division of Allergy, Immunology, Rheumatology Disease, Department of Pediatrics, Mackay Memorial Hospital, Hsinchu, Taiwan; 2 Division of Colorectal Surgery, Department of Surgery, Mackey Memorial Hospital, Taipei, Taiwan; 3 Department of Pediatrics, Mackay Memorial Hospital, Hsinchu, Taiwan; 4 Department of Medical Research, Mackay Memorial Hospital, Taipei, Taiwan; 5 Department of Medicine, Mackay Medical College, No.45, Minsheng Rd., Tamsui Dist., New Taipei City, 25160 Taiwan; 6 Audiology and Speech Language Pathology, Mackay Medical College, No.45, Minsheng Rd., Tamsui Dist., New Taipei City, 25160 Taiwan; 7 Department of Psychiatry, Mackay Memorial Hospital, No.45, Minsheng Rd., Tamsui Dist., New Taipei City, 25160 Taiwan

**Keywords:** Henoch-Scholein Purpura, Recurrent, Incidence, Steroid

## Abstract

**Background:**

The recurrence rate of Henoch-Schönlein purpura (HSP) is 2.7%–30%, with varied average intervals between the first and second episodes. Few studies have explored the incidence and risk factors for recurrent HSP.

**Methods:**

We used a 16-year nationwide database to analyze the incidence of recurrent HSP. Patients with HSP were identified, and risk factors for recurrent HSP were explored. Kaplan-Meier and Cox regression model analyses were performed, and covariates were adjusted in the multivariate model.

**Results:**

From January 1, 1997 to December 31, 2012, among 2,886,836 individuals in the National Health Insurance Research Database, 1002 HSP patients aged < 18 years were identified. Among them, 164 had ≥2 HSP episodes (recurrence rate, 16.4%; incidence of recurrent HSP, 7.05 per 100 person-years); 83.6% patients with one HSP episode remained free of secondary HSP. The average time intervals between the first and second and second and third HSP episodes were 9.2 and 6.4 months, respectively. After adjusting for demographic parameters, comorbidities, and socioeconomic status, recurrent HSP was found to occur more frequently in patients who had renal involvement (adjusted hazard ratio, 2.41; 95% confidence interval [CI], 1.64–3.54; *p* < 0.001), were receiving steroid therapy for > 10 days (adjusted hazard ratio, 8.13; 95%CI, 2.51–26.36; *p* < 0.001), and had allergic rhinitis (adjusted hazard ratio, 1.63; 95%CI, 1.06–2.50; *p* = 0.026).

**Conclusions:**

The annual incidence of recurrent HSP was low. However, children who had underlying allergic rhinitis, presented with renal involvement, and received steroid treatment for > 10 days should be notified regarding the possibility of recurrence.

**Electronic supplementary material:**

The online version of this article (10.1186/s12969-018-0247-8) contains supplementary material, which is available to authorized users.

## Background

Henoch-Schönlein purpura (HSP) is the most common systemic vasculitis in children [1]. The incidence of HSP in children is approximately 6–22 per 100,000 person-years [1–4], which is higher than that in adult (3.4–14.3 per 100,000 person years) [5]. Most HSP symptoms, such as temporarily palpable purpura, gastrointestinal (GI) pain, and joint pain, are self-limited; however, intestinal obstruction, central nervous system involvement, and severe nephritis can also occur [1, 6–10]. The prognosis of HSP is generally good, but recurrence is common among children (recurrence rate, 2.7%–66.2%) [1, 11–16]. Various predictors for recurrence, including greater joint and gastrointestinal involvement at diagnosis, history of infection, elevated erythrocyte sedimentation rate, steroid treatment, and renal manifestations, have been identified but they are inconsistent [1, 12, 13].

Glucocorticoids used for HSP treatment do not prevent renal disease; therefore their use is controversial [17]. However, early treatment with glucocorticoids in HSP children may reduce the intensity or mean resolution time of joint or abdominal pain [18, 19]. Additionally, glucocorticoids should be tapered slowly to prevent the relapse of symptoms. Although previous literature has mainly focused on the risk factors for renal involvement and long-term complications in patients with HSP, no standard protocol or long-term follow-up studies are available to clarify the impact of steroid on the clinical course or subsequent recurrence of HSP.

Furthermore, the few studies that focused on the average time to second episode of recurrence revealed discrepant results (range, 1–13.5 months) [12, 13]. Little is known regarding the third episode of recurrent HSP, and the long-term disease-free rate. However, these data are important in the decision making for adequate follow-up time. To clarify these aspects, data from a nationwide, population-based claims database, the Taiwan National Health Insurance Research Database (NHIRD), were used to investigate the mean duration between first and second HSP episodes, risk factors for recurrence, and the real-world use of steroids in patients with HSP.

## Methods

### Database

On March 1, 1995, the National Health Insurance (NHI) was established in Taiwan, achieving a coverage rate exceeding 99% among Taiwanese citizens. Each year, the Bureau of NHI provides data containing encrypted personal identifications, diagnoses, and healthcare utilizations to compose the Taiwan NHIRD [20]. In the NHIRD, diseases are coded according to the International Classifications of Disease, Ninth Revision, Clinical Modification (ICD-9-CM). A subset of the NHIRD, the Longitudinal Health Insurance Database (LHID), was used in our analysis. The LHID contains 3 million randomly selected enrollees from the NHIRD [21]. Distributions of age, gender, or healthcare costs between the LHID and the general population in Taiwan are not significantly different [22]. This study was reviewed and approved by the institutional Review Board of Mackay Memorial Hospital, Taipei, Taiwan (IRB approval number: 17MMHIS022e). The institutional review board exempted consent requirement.

### Study sample

**Fig. 1:**
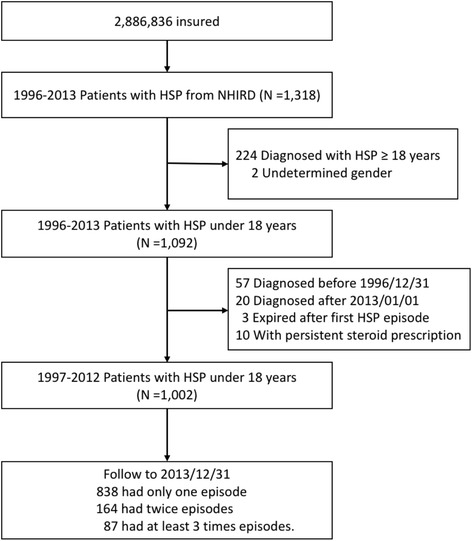
Flow chart of study patients

From the LHID and based on the criteria of the American College of Rheumatology [23], patients aged < 18 years with a first diagnosis of HSP (ICD-9-CM code 287.0) between January 1, 1997, and December 31, 2012, were identified from ambulatory, emergency, and inpatients claims data. Dates of the first HSP were defined as index dates. Patients diagnosed with HSP before December 31, 1996 were excluded. Among these subjects, those who received a second HSP diagnosis 3 months apart from the first HSP diagnosis were defined as having recurrent HSP (study subjects), which means a 3-months diagnosis-free interval between the first and second HSP diagnoses was required in order to define a recurrent HSP. The definition of our 3-months diagnosis-free interval t was based on findings from several epidemiological studies on HSP [12, 14, 24], in which they suggested that HSP commonly recurs within 2 to 3 months after the primary episode. Furthermore, glucocorticoids were suggested to be tapered over a 4 to 8 weeks time frame, to minimize the chance of precipitating a disease flare by overly aggressive medication tapering. Under such considerations, we found and excluded 10 patients with persistent HSP diagnosis and persistent steroid prescription in every follow-up outpatient visit during and after 3 months time because they may be seen as receiving continued treatment, instead of having relapse or recurrence. All patients included as recurrent HSP had records of HSP diagnosis-free interval for at least 3 months after the index date. Figure 1 shows the flow diagram of participant selection. Status and durations for steroid use (solu-medrol, solu-cortef, kidsolone, and prednisolone) were compared. All the generic names of steroid included in our analysis were attached in the supplement Additional file 1: Table S1. Corticosteroid exposure was characterized by the day of initial prescription and the duration of treatment. Children were divided into the following categories based on the time of initiating corticosteroids: (1) first dose prescribed within < 14 days of initial diagnosis,(2) first dose prescribed after day 14 of initial diagnosis, and (3) no receipt of steroids during the disease course. Fourteen days was chosen as the cutoff point for corticosteroid initiation to include patients with atypically late presentations or those with later worsened clinical course. The duration of corticosteroid use was defined as consecutive days of corticosteroids administration [25]. Currently, the recommended steroid treatment duration is 1–2 weeks for HSP [18]. However, there was no precise definition about the length of steroid therapy. So, we chose the median number of days in steroid use among our study subjects as the cutoff point to group patients with steroid use. For the sake of brevity, patients were categorized into those that used steroids for < 10 days (the shorter-term steroid use group); and ≥ 10 days (the longer-term steroid use group), respectively. HSP with renal involvement was defined as receiving a diagnosis of hematuria, proteinuria, nephritis, or nephrotic syndrome (ICD-9-CM codes 599.7, 791.0, and, 580–585) within 1 year after the first HSP diagnosis. Renal biopsy was defined by the ICD-9-CM codes 55.23 (Closed (percutaneous) (needle) biopsy of kidney) and 55.24 (Open biopsy of kidney)). Other clinical manifestations for HSP were defined as having the following diagnoses: joint pain (ICD-9-CM code 719.4×) or GI symptoms (ICD-9-CM codes 789, 787.0, 787.91, 558.9, and 578.9). Our covariates included demographic data (e.g., age, gender, and income levels) and diagnoses of asthma (ICD-9-CM code 493 and 494), allergic rhinitis (ICD-9-CM code 477.x), and atopic dermatitis (ICD-9-CM codes 691.8), which appeared before the diagnosis of the first HSP. All subjects were followed until December 31, 2013.

### Statistical analysis

Data analysis was conducted using SPSS 18.0 software for descriptive and contingency tables (SPSS, Inc., Chicago, Illinois). Pearson’s chi-squared tests were used for comparisons of categorical variables. The Kruskal-Wallis test was used to investigate average differences in age at HSP diagnosis and/or recurrence. Kaplan-Meier survival analysis and Cox proportional hazard models were used to estimate hazard ratios and adjust for other covariates. The event was the date of HSP recurrence. Censoring points were the end of follow-up or the date of withdrawal from the registry. *P* < 0.05 was considered statistically significant. This study was approved by the institutional review board of Mackay Memorial Hospital (IRB no: 17MMHIS022e).

## Result

### Recurrent rate and incidence

We identified 1002 patients aged < 18 years with a first diagnosis of HSP (index episode; Table 1; 50.8% females). Nearly 59.5% had HSP before 6 years of age, nearly one-fifth had recurrent events (secondary HSP), and nearly 10% ≥3 episodes of HSP. Recurrent HSP patients were predominantly male and younger aged (second recurrent HSP episode before 6 years of age). Furthermore, the third HSP episode occurred in higher proportions of individuals aged 7–12 years.

**Table 1:** Henoch-Schönlein Purpura and recurrent events (*N* = 1002)

	One HSP episode (*n* = 838) n (%)	Two HSP episodes (*n* = 164) n (%)	Three HSP episodes (*n* = 87) n (%)
Gender			
Female	426 (50.8)	73 (44.5)	41 (47.1)
Male	412 (49.2)	91 (55.5)	46 (52.9)
Age, y			
0–6	499 (59.5)	82 (50.0)	37 (42.6)
7–12	268 (32.0)	71 (43.3)	43 (49.4)
13–18	71 (8.5)	11(6.7)	7 (8.0)

Abbreviations: *HSP* Henoch-Schönlein Purpura

The incidence of the first HSP episode was 9.61per 100,000 person-years (male incidence, 4.82; female incidence, 4.79). The highest incidence (5.45 per 100,000 person-years) of first HSP was among the youngest age group (0–6 years).

The incidence of patients with secondary (recurrent) HSP was 7.05 per 100 person-years (male incidence, 8.29; female incidence, 4.96). The highest incidence (11.03 per 100 person-years) of recurrent HSP was among the youngest age group (0–6 years), and the lowest (2.87 per 100 person-years) was among the oldest age group (13–18 years).

### Average time of steroid usage

Among the 1002 HSP patients, 342 (40.8%) used steroids during the first HSP episode. The average period of steroid use during the first HSP episode was 6.90 days. Among the 77 patients with a second HSP episode, 43 (55.8%) received steroid therapy. The average period of steroid use during the second HSP episode was 10.09 days. Finally, among the 27 patients with a third HSP episode, 20 (74.1%) used steroids. The average period of steroid use during the third episode was 16.30 days. The percentage and duration of steroid use in each episode was listed in Additional file 2: Table S2.

### Mean duration between first and second HSP episodes

**Fig. 2:**
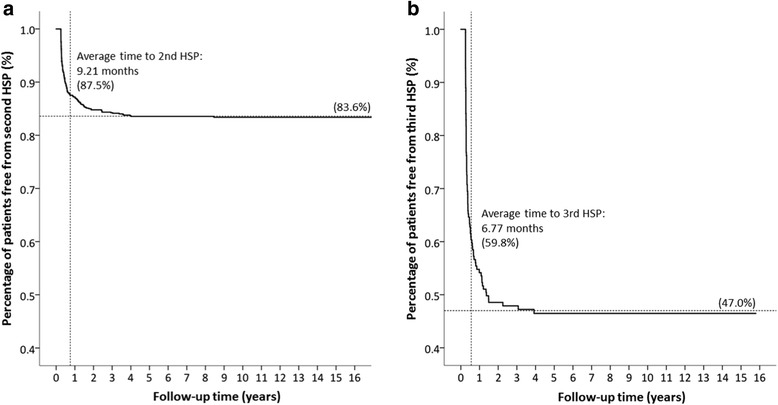
Kaplan-Meier analysis. **a**, at the end of the 16-year cohort, 83.6% of patients remained free of a second HSP episode. The mean time between the first and the second HSP episodes was 9.21 months. **b**, among patients with second HSP, 47.0% of patients remained free of the third HSP episode. The average time from the second to the third HSP episode was 6.77 months

The mean duration between first and second HSP episodes was 9.21 months (Fig. 2a); 47.0% patients with second HSP did not have a third HSP episode. The average time between the second and third HSP episodes was 6.77 months (Fig. 2b).

### Influence of age, clinical course, and steroid on the incidence of secondary HSP

Table 2 lists the hazard ratios for recurrent HSP obtained from univariate Cox proportional analysis. Compared with patients aged < 6 years, patients aged 7–12 years were more likely to have a second HSP episode. Recurrent HSP episodes were more likely to occur in those with underlying allergic rhinitis and renal involvement during the first HSP episode. Compared with patients who did not use steroids, patients who underwent steroid therapy for 14 days after the HSP diagnosis (late initiation) were more likely to have HSP recurrence. Furthermore, patients who used steroids for > 10 days had a higher likelihood of experiencing a second HSP episode. Joint pain and gastrointestinal symptoms during the first HSP course were not associated with a second HSP episode. Income levels and other comorbidities had no significant impact on the incidence of secondary HSP.

**Table 2:** Henoch-Schönlein Purpura events. (N = 1002)

	Only one Episode (n = 838), n (%)	Recurrent Episode (n = 164), n (%)	Crude HR (95% CI)^*b*^	*p* value^∮^
Sex				
Male	412 (49.2)	91 (55.5)	1.27(0.93–1.73)	0.131
Female	426 (50.8)	73 (44.5)	1.00	
Age, y				
Mean ± SD	6.85 ± 3.70	7.22 ± 3.37	1.03 (0.99–1.07)	0.224
0–6	499 (59.5)	82 (50.0)	1.00	
7–12	268 (32.0)	71 (43.3)	1.55 (1.13–2.13)	0.007
13–18	71 (8.5)	11 (6.7)	0.95 (0.51–1.79)	0.880
Economics Status				
Normal	771 (92.0)	154 (93.9)	1.00	
Low income	67 (8.0)	10 (6.1)	0.77 (0.40–1.45)	0.411
Comorbidities				
Asthma	356 (42.5)	68 (41.5)	0.97 (0.71–1.33)	0.866
Allergic rhinitis	627 (74.8)	139 (84.8)	1.80 (1.18–2.77)	0.006
Atopic dermatitis	221 (26.4)	47 (28.7)	1.13 (0.80–1.58)	0.484
Clinical course				
GI symptoms	799 (95.3)	161 (98.2)	2.50 (0.80–7.82)	0.116
Renal symptoms	57 (6.8)	36 (22.0)	3.15 (2.17–4.56)	< 0.001
Joint Pain	111 (13.2)	30 (18.3)	1.39 (0.94–2.07)	0.102
Initiation of Steroid^(1st)^				
No steroid use	496 (59.2)	67 (40.9)	1.00	
Early (< 14 days)	19 (2.3)	4 (2.4)	1.50 (0.55–4.12)	0.430
Late (≥ 14 days)	323 (38.5)	93 (56.7)	1.97 (1.44–2.70)	< 0.001
Duration of Steroid				
No steroid use	496 (59.2)	67 (40.9)	1.00	
SG (< 10 days)	284 (33.9)	48 (29.3)	1.20 (0.83–1.74)	0.329
LG(≥ 10 days)	58 (6.9)	49 (29.9)	4.98 (3.45–7.21)	< 0.001

Abbreviations: *HSP* Henoch-Schönlein Purpura, *SD* standard deviation, *SG* short-term group, LG long-term group, *HR* Hazard ratio

^∮^Cox proportional Regression Analysis

^b^Hazard ratio of 1.00 indicates reference group

**Fig. 3:**
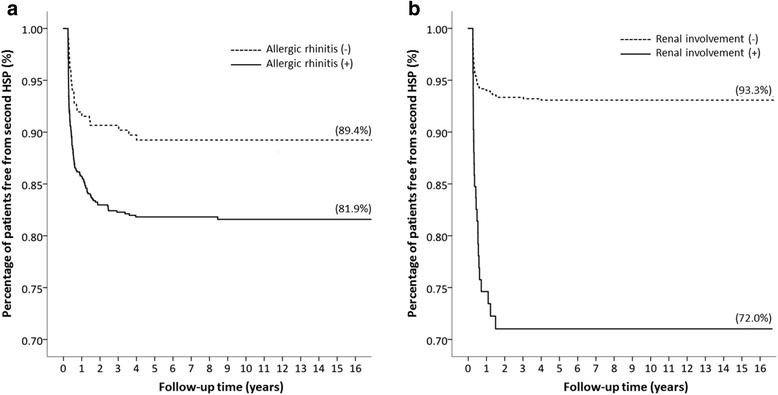
Allergic rhinitis and renal involvement-specific Kaplan-Meier analysis. **a**, at the end of the 16-year cohort, the percentage of patients free of a second HSP episode was 89.4% in children without allergic rhinitis and 81.9% in those with allergic rhinitis. **b**, the percentage was 93.3% and 72.0% for patients without and with renal involvement, respectively

**Fig. 4:**
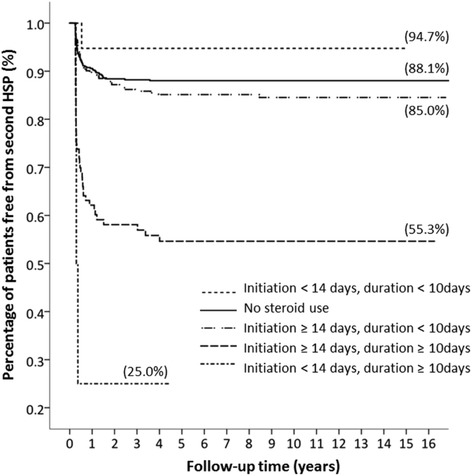
Kaplan-Meier analysis according to the initiation and duration of steroid use. At the end of the 16-year cohort, the percentage of patients free of a second HSP was 94.7%, 88.1%, 84.0%, 55.3%, and 25.0% for patients with steroid initiation < 14 days and duration < 10 days, no steroid use, initiation ≥14 days and duration < 10 days, initiation ≥14 days and duration ≥10 days, initiation < 14 days and duration ;≥10 days, respectively

After adjusting for age, gender, and medical comorbidities, increased associations of renal involvement, underlying allergic rhinitis, and longer steroid therapy with the recurrence of HSP were still noted (Table 3). At the end of the 16-year cohort, patients without allergic rhinitis had less recurrent episodes than those with underlying allergic rhinitis (89.4% and 81.9% were free of recurrence, respectively; *P* = 0.026; Fig. 3a). A second HSP episode occurred in 93.3% patients without renal involvement and 72.0% patients with renal involvement(Fig. 3b). Patients with only the first HSP episode were further stratified into five subgroups according to the initiation and duration of steroid therapy (Fig. 4). Percentages of patients without second HSP were 88.1% for the non-steroid group, 94.7% for the subgroup with steroid initiation within 14 days of diagnosis and steroid duration < 10 days, 85.0% for the subgroup with steroid initiation at ≥14 days after diagnosis and steroid duration < 10 days, 55.3% for the subgroup with steroid initiation at ≥14 days after diagnosis and steroid duration ≥10 days, and 25.0% for the subgroup with steroid initiation within 14 days of diagnosis and steroid duration ≥10 days (Fig. 4). Longer-term steroid use was associated with recurrent HSP regardless of early (< 14 days) or late (≥14 days) steroid initiation (*P* < 0.001, log-rank test).

**Table 3:** Risk analysis of a second (Recurrent) Henoch-Schönlein Purpura episode

	Adjusted HR (95%CI)^*b*^	*p* value
Demographic factors		
Age, y	1.01 (0.96–1.05)	0.791
Comorbidities		
Allergic rhinitis	1.63 (1.06–2.50)	0.026
Clinical course		
Renal symptoms	2.41 (1.64–3.54)	< 0.001
Steroid initiation and duration^(1st)^		
No use record	1.00	
Initiation < 14 days and Duration ≤ 10 days	0.40 (0.06–2.90)	0.366
Initiation < 14 days and Duration > 10 days	8.13 (2.51–26.36)	< 0.001
Initiation ≥ 14 days and Duration ≤ 10 days	1.21 (0.83–1.76)	0.321
Initiation ≥ 14 days and Duration > 10 days	4.06 (2.76–5.96)	< 0.001

^b^Hazard ratio of 1.00 indicates reference group

## Discussion

To our knowledge, this is the first population-based cohort study investigating the incidence and risk factors of recurrent HSP. Our results revealed that renal involvement, underlying allergic rhinitis, and steroid treatment for > 10 days were risk factors for HSP recurrence in children, regardless of age, sex, and income levels.

The recurrence rate (16.3%) in our study was within the range described by a previous study (2.7%–66.2%) [1, 12–16]. Reasons for discrepancies between our results and previous research may be partly related to different definitions of recurrence and patient selection. In this study, we defined our recurrent events as being re-diagnosed 3 months apart from the first HSP, which was also used by previous research, including one epidemiological HSP study in Taiwan [12, 14, 24]. Patients with a second diagnosis of HSP from both outpatient and inpatient departments were included in our study, whereas in previous studies, recurrence rates may have been underestimated if the definition of recurrent HSP was limited to patients readmitted for treatment [12, 16], because hospitalization is not always necessary for HSP patients unless complications (such as dehydration, severe abdominal pain, gastrointestinal bleeding, joint pain with ambulatory limitations, and renal insufficiency) occur. Conversely, recurrence may have been overestimated in studies that defined their recurrent episode as after at least 2–4 weeks of asymptomatic periods, because relapses of symptoms may occur after 4–6 weeks of spontaneous resolution [26]. Additionally, studies that defined recurrent episodes as asymptomatic for at least 2–4 weeks had the highest recurrent rate (33%–66%) [1, 7, 13, 15]. However, in children with HSP, relapses of symptoms occurred over 4–6 weeks before spontaneous resolution even in the absence of a complicated disease course [27].Thus, remission of symptoms for 2–4 weeks may not be adequate for HSP patients to affirm recovery. Furthermore, steroid therapy use may be another explanation for different recurrence rates between studies. In our study, the proportion of steroid use in first HSP was 40.8%. There might be an association between more steroid use and lower rates of recurrence. Lee et al. described a low recurrence rate of 5.2%, and the majority of patients (88.7%) from their study were treated with steroids [28]. Fretzayas et al. applied the strictest policy of using corticosteroids only in 18% of patients with a severe clinical course and reported the highest recurrence rate (66%) to date [15]. The choice of using steroids may be confounded by the disease severity. However, there was no significant difference in the initial clinical severity between the steroid-treated and non-steroid treated groups in Lee’s report. Nevertheless, such observation requires further validation in different populations.

Our finding that allergic rhinitis was an independent risk factor for HSP was in line with studies reporting an increased risk of HSP in atopic children [29, 30]. However, no study has addressed the correlation between recurrent HSP and allergic diseases. For instance, elevated serum Th2-related cytokine levels (such as interleukin [IL]-4 and IL-5, and IgE) were reported in children with HSP [31–34]. Most HSP occurred after bacterial or viral infections, insect bites, or even food allergies, suggesting that allergic reactions predispose to HSP initiation [26, 35]. HSP is characterized by elevated serum IgA levels and vascular deposition of IgA immune complex, causing vascular necrosis mediated by IgE-sensitized mast cells [33]. For those with allergic rhinitis, the release of Th2-related cytokines results in chronic mucosal inflammation [36]. HSP patients with underlying allergic rhinitis may be more susceptible to chronic inflammatory status leading to a more intense immune response to causative antigens, thereby predisposing the recurrence of HSP. Moreover, the nasal mucosa (other than the bronchiole epithelium) is the first line of defense against pathogens [37]. The inability of the ailing mucosa to remove pathogens could forecast an infectious disease, which is a critical predisposing causative factor for HSP. This may partly explain why allergic rhinitis other than asthma or atopic dermatitis was a risk factor for recurrent HSP in this study. Further studies are needed to clarify the pathogenesis of this association.

Regarding the clinical manifestations of HSP, renal involvement remains a major concern because it may lead to permanent renal function impairment. In conformity with Jauhola et al. and Alfredo et al., who both reported higher recurrences in patients with nephritis but not in those with joint symptoms and GI manifestations [14, 38], our result revealed a higher risk of recurrence in patients with renal involvement (HR, 2.41). Additionally, the average time to recurrent episode in our patients with HSP nephritis was also shorter. Nevertheless, HSP nephritis affected 6.8% of patients in the current study, which is lower than the 10%–50% reported in previous literature [39] Moreover, our result that HSP nephritis occurred in 22% of patients with a recurrent episode was also lower than the 58.3% reported by Alfredo et al. [14]. Although the pathogenic mechanisms of HSP nephritis remain unclear, studies have described that galactose-deficient IgA1, recognized by antiglycan antibodies, might lead to the formation of circulating immune complexes and their mesangial deposition, resulting in renal injury among HSP patients [7].

Our study showed a significantly increased association between steroid use for > 10 days regardless of early initiation and the risk of recurrence. Steroids might have some treatment effects on HSP through their anti-inflammatory ability. Although early steroid therapy can ameliorate acute abdominal symptoms and mitigate short-term morbidity [18, 40–43], previous literature revealed conflicting results on the association between corticosteroid treatment and recurrence. Pamela et al. reported a non-significant protective effect of corticosteroids on recurrence rate [19]. However, Trapani et al. and Calvo-Rio et al. observed an opposite finding that emphasized an increased association between early corticosteroid use and the risk of relapse [1, 13]. In Trapani’s case series of 150 patients, steroids were prescribed only in patients with a severe presentation, including 16 patients with severe abdominal and 3 patients with severe nephropathy. Their results of increased association might be explained by the fact that patients prescribed with corticosteroids had a more severe disease manifestation [1, 44], which is a risk factor for recurrence [44]. Currently, steroid therapy is suggested only in patients with nephritis and severe GI symptoms (dosage, 1–2 mg/kg/day). One might argue that our results of elevated recurrence rates in those with steroid use for > 10 days were associated with their disease severity. However, this may only partly explain our result because not all recurrences were associated with more severe clinical courses, such as renal involvement. Shin et al. also pointed out that there is a distinct group of multiple recurrent non-renal HSP [45]. Our findings indicate that the risk of recurrence might be associated with a longer duration of steroid use in certain patients. Thus, we hypothesized the mechanism that the longer use of steroid might indicate an “over-prescribing” of steroid, which might interfere with the clinical course, including the chance of recurrence. To date, there is no optimal recommendation for the duration of treatment. Expert opinions have suggested that steroids should be tapered slowly by < 25% per week to prevent relapse, thus, the overall duration of steroid treatment may easily exceeds 10 days and generally requires 4 weeks. Since the optimal duration and timing of steroid administration remains unanswered, further prospective randomized trials are needed to elucidate this possible underlying mechanism between longer-term steroid use and HSP recurrences.

The duration of follow-up in children with HSP for the early identification of possible recurrence is an important issue. In our study, the average duration between the first and second HSP episodes was 9.2 months (range, 4 months to 1 year in previous literature) [1, 8, 46]. Prais et al. described a longer duration of 13.5 months between episodes [12]. The reason for the longer period in Prais’s study may be because they only considered recurrences that required hospitalization in their analysis. We were the first study to report a 6.4-month average duration between the second and the third recurrences. Although there were only 6% of children with > 3 recurrences during our follow-up period, nearly half of the patients with second HSP had a third HSP event. Thus, for the early detection of recurrence, it may be worth considering following up patients with the first HSP episode for at least 9.2 months and those with a second HSP episode for another 6.4 months.

### Strengths and limitations

A strength of the current study is the use of a nationwide, population-based dataset large enough to provide sufficient statistical power for detecting the association of interest. The distinctive feature of the data source strengthens the validity of the estimated disease incidences [47]. However, there were still several limitations in this study. First, although we have adjusted for covariates such as age, sex, income levels, and comorbidities, we were unable to control for information not recorded in the NHIRD, such as serum IgE and specific IgE levels, eosinophil counts, levels of albumin, triglyceride, urine protein, urine creatinine, infection pathogens, or genetic factors. Second, this population- based study mostly assessed the Han Chinese ethnic group, and the findings may not be generalized to other populations. Third, a proper definition of ‘recurrence’ was supposed to be defined as the occurrences of new symptoms after an initial remission, requiring the resumption of immunosuppressive therapy or an increased dose. Unfortunately, details of symptoms were not recorded in the NHIRD database. Besides, not every patient with HSP needs hospitalization or was prescribed with medications. As a consequence, we were unable to use symptoms or steroid prescription to define the disease episode or disease free intervals. However, the definition of our recurrent events as at least 3-months apart from the first HSP diagnosis was in accordance to previous research, and also considered the duration of tapering steroid. Although patients that never remitted from their first episode and not treated in the NHI system between the two diagnoses 3- months apart might be included as recurrent subject, such chances were small because over 99% of the citizens utilizes health care through the NHI program.

## Conclusion

Ours was the first study to describe the incidence of recurrent HSP, and added evidence of increased associations of recurrent HSP and allergic rhinitis, renal involvement, and steroid treatment for > 10 days. These results can be considered observations from real-world conditions. Patients with the aforementioned clinical features were suggested to receive longer periods of follow-up than previously suggested for the early identification and management of recurrent HSP. In those with a second HSP episode, perhaps 6 more months of monitoring is also suggested for the early recognition of a third HSP recurrence. Further study is needed to clarify the underlying pathogenic mechanisms of these associations.

## Additional files

Additional file 1: **Table S1.** The generic name of steroid used in current cohort. (DOCX 133 kb)

Additional file 2: **Table S2.** The percentage and duration of steroid use in each episode. (DOCX 18 kb)

## Abbreviations

CI: Confidence interval
GI: Gastrointestinal
HR: Hazard ratio
HSP: Henoch-Schönlein purpura
IgA: Immunoglobulin A
IgE: Immunoglobulin E
IL: Interleukin
LG: Long-term group
LHID: Longitudinal Health Insurance Database
NHI: National Health Insurance
NHIRD: Taiwan National Health Insurance Research Database
SD: Standard deviation
SG: Short-term group
Th1: T helper cell 1
Th2: T helper cell 2
