# Supplementary material for: Incidence and risk factors for recurrent Henoch-Schönlein purpura in children from a 16-year nationwide database
Source: Pediatr Rheumatol Online J. 2018 Apr 16;16:25. doi: 10.1186/s12969-018-0247-8 (PMC5902957; doi:10.1186/s12969-018-0247-8)
Supplement: Supplementary file 1 — Table S1. The generic name of steroid used in current cohort. (DOCX 133 kb) [file 12969_2018_247_MOESM1_ESM.docx]

| **Table S1 The generic name of steroid used in current cohort** |
| --- |
| CORTISONE ACETATE TABLETS 25MG "PING LAWER" |
| SOLU-TISONE INJECTION 100MG "C.C.P." (HYDROCORTISONE) |
| SOLU-TISONE INJECTION 100MG "C.C.P." (HYDROCORTISONE) |
| SAXIZON INJECTION (HYDROCORTISION SUCCINATE) "SHUN HWA" |
| SAXIZONPOWDER FOR INJECTION 300MG |
| CORTISONE ACETATE TABLETS 25MG "WEAL-CHANCE" |
| HYSON POWDER FOR INJECTION |
| HYDROCORTISONE POWDER FOR INJECTION 100MG"S.T." |
| CORTISONE ACETATE TABLETS 25MG "PING LAWER" |
| CORTISONE ACETATE TABLETS 25MG “PINE LAWER”(鋁箔/膠箔) |
| SOLU-TISONE INJECTION 100MG "C.C.P." (HYDROCORTISONE) |
| CORTISONE ACETATE TABLETS 25MG "WEAL-CHANCE" |
| CORTISONE ACETATE TABLETS 25MG "WEAL-CHANCE"(鋁箔/膠箔) |
| HYDROCORTISONE POWDER FOR INJECTION 100MG"S.T." |
| HYDROCORTISONE INJECTION 100MG "CYH" |
| SOLU-CORTEF 100MG |
| SOLU-CORTEF INJECTION 250MG |
| SOLU-CORTEF INJECTION 500MG |
| SAXIZON INJECTION 300MG |
| SAXIZON INJECTION 100MG |
| WATER-SOLUBLE SUCCORT INJECTION |
| SAXIZON INJECTION 500MG |
| HYDROCORTISONE ACETATE AQUOSUSP. INJECTION "FUJI" |
| UPASE AMPOULES |
| FLORINEF TABLETS 0.1MG |
| SOLU-CORTEF STERILE POWDER |
| SOLU-CORTEF STERILE POWDER |
| SOLU-CORTEF STERILE POWDER 100MG |
| SOLU-CORTEF 100MG |
| FLORINEF TABLETS 0.1MG |
| SOLU-CORTEF STERILE POWDER 100MG |
| FLORINEF TABLETS 0.1MG |
| CORTISONE TABLETS |
| CORTISONE TABLETS |
| CORTEMA ENEMA 1.667MG/ML "PURZER" (HYDROCORTISONE) |
| PREDNISOLONE TABLETS " NAN YA" |
| PIDONIN TABLETS "N.T." |
| PREDNISOLONE TABLETS 5MG "S.Y." |
| PREDNISOLONE TABLETS "YU SHENG" |
| PREDNISOLONE TABLETS "SANTONG" |
| PREIN TABLETS "T.F." |
| PREDNISOLONE TABLETS 5MG "VPP" |
| PREDNISOLONE TABLETS 5MG "VPP"(鋁箔/膠箔) |
| PREDNISLOLNE TABLETS "TON SHENG" |
| PREDNISOLONE TABLETS "ASTAR" |
| PREDNISOLONE TABLETS "DAU CHI" |
| PREDNISOLONE TABLETS 5MG "CHEN TA" |
| PUN LIH LONG TABLETS "L.D." |
| PREDNISOLONE TABLETS "M.S." |
| PRENINE TABLETS |
| PREDNISOLONE TABLETS "P.L." |
| PRENIN TABLETS |
| SUNPRED TABLETS |
| PREDNISOLONE TABLETS "YUNG CHI" |
| PREDSON TABLETS "H.S." |
| DONISON INJECTION 25MG |
| PREDNISOLONE TABLETS "MEIDER" |
| PREDNISOLONE TABLETS "C.R" |
| PREDNISOLONE TABLETS "C.H." |
| PREDNISOLONE TABLETS "C.I." |
| PREDNISOLONE TABLETS "WINSTON" |
| PREDNISOLONE TABLETS "WINSTON"（鋁箔/膠箔） |
| PRELONE TABLETS (PREDNISOLONE) "K.B." |
| PREDNISOLONE TABLETS "P.J." |
| PREDNISOLONE TABLETS "L.S." |
| PREDNISOLONE TABLETS 5MG "C.T." |
| PRESON TABLETS (PREDNISOLONE) "VEIMIN" |
| LYO-MEDONIN INJECTION 500MG "C.C.P." |
| PROCOSONE TABLETS (PREDNISOLONE) "KODAK" |
| SIULON TABLETS |
| MEDASON FOR INJECTION 500MG (METHYLPREDNISOLONE)"N.K." |
| MEDASON FOR INJECTION |
| MEDASON FOR INJECTION |
| MEDASON FOR INJECTION |
| MEDASON FOR INJECTION |
| METISONE TABLETS 2MG "S.T." (METHYLPREDNISOLONE) |
| PREDNISOLONE TABLETS 5MG |
| CHANSIAN CAPSULES 5MG (PREDNISOLONE) "HS" |
| COMPESOLON TABLETS 5MG "PINE LAWER" (PREDNISOLONE) |
| COMPESOLON TABLETS 5MG "PINE LAWER" (PREDNISOLONE) (鋁箔/膠箔) |
| DELTA-CORTEF TABLETS 5MG (PREDNISOLONE) |
| PRITHMOW TABLETS 5MG (PREDNISOLONE)"Y.C." |
| PREDNISOLONE S.C. TABLETS 5NG "F.S." |
| ANTHTON-P CAPSULES |
| PREDNISOLONE 5MG TABLETS "Y.Y." |
| PREDNISOLONE 5MG TABLETS "Y.Y." (鋁箔/膠箔) |
| PREDNISOLONE 5MG TABLETS "Y.Y."(84粒/瓶) |
| PRECONIN TABLETS 5MG (PREDNISOLONE)"M.S." |
| MEDNIN TABLETS 4MG (METHYLPREDNISOLONE)"H.S." |
| MEDLIN TABLETS 4MG (METHYLPREDNISOLONE) "M.S." |
| METHYLPREDNISOLONE TABLETS 4MG "YUNG SHIN" |
| METISONE TABLETS 4MG (METHYLPREDNISOLONE) "S.D." |
| MEPRED TABLETS 4MG (METHYLPREDNISOLONE) "F.S." |
| PESOLONE TABLETS 5MG (PREDNISOLONE) "CENTER" |
| PREA TABLETS 4MG (METHYLPREDNISOLONE) "Y.C." |
| MESURIN TABLETS 2MG"S.C"(METHYLPREDNISOLONE) |
| MEDLIN TAB. 4MG |
| MEDLIN TABLETS 4MG "KOJAR" (METHYLPREDNISOLONE) (鋁箔/膠箔) |
| PRECONIN TABLETS. 5MG"KOJAR" (PREDNISOLONE) |
| PRECONIN TABLETS 5MG (PREDNISOLONE) "KOJAR"(鋁箔/膠箔) |
| PREDNISOLONE TABLETS 5MG |
| MENISONE TABLETS 4MG (METHYLPREDNISOLONE) "P.L." |
| MEPSON TABLETS 8MG "SHIN FONG" (METHYLPREDNISOLONE) |
| MELIN TABLETS 4MG "H.C." (METHYLPREDNISOLONE) |
| MELONE TABLETS 16MG |
| MESOLONE TABLETS 2MG "H.C" |
| MEPRON POWDER FOR INJ.１G "GENTLE" |
| MEPRON POWDER FOR INJECTION 0.04GM "GENTLE" |
| MEPRON POWDER FOR INJ. 0.25G "GENTLE" |
| MEPRON INJ 0.5G "GENTLE" |
| MEDNIN TABLETS 2MG (METHYLPREDNISOLONE) "H.S" |
| METHYLONE TABLETS 4MG (METHYLPREDNISOLONE) |
| EXCELIN TABLETS 4MG (METHYLPREDNISOLONE) "WINSTON." |
| MEP TABLET |
| PRENILONE TABLETS 5MG "TAI YU" (PREDNISOLONE) |
| BELON INJECTION 40MG "ORIENTAL" |
| METICORT TABLETS 2MG "KINGDOM" |
| METICORT TABLETS 4MG "KINGDOM" (METHYLPREDNISOLONE) |
| MESOLONE TABLETS "Y.C." |
| PREDNISOLONE TABLETS 5MG "WEAL-CHANCE"(鋁箔/膠箔) |
| KIDSOLONE ORAL SOLUTION "CENTER" |
| KIDSOLONE ORAL SOLUTION "CENTER" |
| KIDSOLONE ORAL SOLUTION "CENTER" |
| SHINMING TABLETS 4MG "TAI YU" |
| MENISON TABLETS 2MG "P.L" |
| METISOL INJECTION |
| BONY Tablets "Y.C." |
| LICOLONE TABLETS 4MG "ASTAR" |
| Methylprednisolone Injection 500mg "Yung Shin" |
| Methylprednisolone Injection 40mg "Yung Shin" |
| MeHo Tablets "Y.C." |
| BERON F.C.TABLETS 8MG“EVEREST” |
| POLYNISOLONE ORAL SOLUTION "GS" |
| POLYNISOLONE ORAL SOLUTION "GS" |
| POLYNISOLONE ORAL SOLUTION "GS" |
| COLON TABLETS "D.T.S" |
| PREDNISOLONE TABLETS "WINSTON" |
| PRECONIN TABLETS. 5MG"KOJAR"(PREDNISOLONE) |
| PRECONIN TABLETS 5MG (PREDNISOLONE) "KOJAR"(鋁箔/膠箔) |
| PREDNISOLONE TABLETS "YU SHENG" |
| PREIN TABLETS "T.F." |
| PREDNISOLONE TABLETS 5MG "VPP" |
| PREDNISOLONE TABLETS 5MG "VPP"(鋁箔/膠箔) |
| "HEALTH" PREDINISOLONE TABLETS |
| "HEALTH" PREDINISOLONE TABLETS(鋁箔/膠箔) |
| "PREDNISOLONE TABLETS ""ASTAR""" |
| PREDNISOLONE TABLETS "P.L." |
| PREDNISOLONE TABLETS |
| PREDNISOLONE TABLETS(鋁箔/膠箔) |
| PREDSON TABLETS "H.S." |
| PREDNISOLONE TABLETS "MEIDER" |
| PREDNISOLONE TABLETS "C.R" |
| PREDNISOLONE TABLETS "C.H." |
| PREDNISOLONE TABLETS "P.J." |
| MEDASON FOR INJECTION |
| MEDASON FOR INJECTION |
| MEDASON FOR INJECTION |
| MEDASON FOR INJECTION |
| METISONE TABLETS 2MG "S.T." (METHYLPREDNISOLONE) |
| PREDNISOLONE TABLETS 5MG |
| PREDNISOLONE TABLETS 5MG(鋁箔/膠箔) |
| "CHANSIAN CAPSULES 5MG (PREDNISOLONE) ""HS""" |
| COMPESOLON TABLETS 5MG "PINE LAWER" (PREDNISOLONE) |
| COMPESOLON TABLETS 5MG "PINE LAWER" (PREDNISOLONE) (鋁箔/膠箔) |
| PRITHMOW TABLETS 5MG (PREDNISOLONE)"Y.C." |
| PREDNISOLONE 5MG TABLETS "Y.Y." |
| PREDNISOLONE 5MG TABLETS "Y.Y." (鋁箔/膠箔) |
| PREDNISOLONE 5MG TABLETS "Y.Y."(84粒/瓶) |
| MEDNIN TABLETS 4MG "H.S." (METHYLPREDNISOLONE) |
| METHYLPREDNISOLONE TABLETS 4MG "YUNG SHIN" |
| METHYLPREDNISOLONE TABLETS 4MG "YUNG SHIN"(鋁箔/膠箔) |
| METISONE TABLETS 4MG (METHYLPREDNISOLONE) "S.D." |
| PREA TABLETS 4MG (METHYLPREDNISOLONE) "Y.C." |
| MESURIN TABLETS 2MG"S.C"(METHYLPREDNISOLONE) |
| MEDLIN TAB. 4MG |
| MEDLIN TABLETS 4MG "KOJAR" (METHYLPREDNISOLONE) (鋁箔/膠箔) |
| PRECONIN TABLETS. 5MG"KOJAR"(PREDNISOLONE) |
| PRECONIN TABLETS 5MG (PREDNISOLONE) "KOJAR"(鋁箔/膠箔) |
| MENISONE TABLETS 4MG (METHYLPREDNISOLONE) "P.L." |
| MELONE TABLETS 16MG |
| MESOLONE TABLETS 2MG "H.C" |
| MEPRON POWDER FOR INJ.１G "GENTLE" |
| MEPRON POWDER FOR INJECTION 0.04GM"GENTLE" |
| MEPRON POWDER FOR INJ. 0.25G "GENTLE" |
| MEPRON INJ. 0.5G "GENTLE" |
| MEDNIN TABLETS 2MG (METHYLPREDNISOLONE) "H.S" |
| METHYLONE TABLETS 4MG (METHYLPREDNISOLONE) |
| "EXCELIN TABLETS 4MG (METHYLPREDNISOLONE) ""WINSTON.""" |
| MEP TABLET |
| "PRENILONE TABLETS 5MG ""TAI YU"" (PREDNISOLONE)" |
| METICORT TABLETS 4MG "KINGDOM" (METHYLPREDNISOLONE) |
| MESOLONE TABLETS "Y.C." |
| PREDNISOLONE TABLETS 5MG "WEAL-CHANCE"(鋁箔/膠箔) |
| KIDSOLONE ORAL SOLUTION "CENTER" |
| KIDSOLONE ORAL SOLUTION "CENTER" |
| KIDSOLONE ORAL SOLUTION "CENTER" |
| "SHINMING TABLETS 4MG ""TAI YU""" |
| MENISON TABLETS 2MG "P.L" |
| BONY Tablets "Y.C." |
| Methylprednisolone Injection 500mg "Yung Shin" |
| Methylprednisolone Injection 40mg "Yung Shin" |
| MeHo Tablets "Y.C." |
| PREDNICONE OPHTHALMIC SUSPENSION 1 % |
| BERON F.C.TABLETS 8MG“EVEREST” |
| ANTIFLAM ORAL SOLUTION "CHI SHENG" |
| PREDNISOLONE TABLETS 5MG "CYH" |
| PREDNISOLONE TABLETS 5MG "CYH"(鋁箔/膠箔) |
| COLON TABLETS "D.T.S" |
| PREON TAB |
| SOLU-MEDROL STERILE POWDER 40MG |
| SOLU-MEDROL STERILR POWDER 125MG |
| STERILE SOLU-MEDROL 500MG |
| SINTISONE TABLETS |
| URBASON (R) SOUBILE FORTE 1000 FOR INJECTION |
| URBASON (R) SOUBILE FORTE 1000 FOR INJECTION |
| SOLU-MEDROL 2 GRAM STERILE POWDER |
| MEDROL TABLETS 100MG |
| HEMICORT 40 MG |
| HEMICORT 125 MG |
| HEMICORT 2 G |
| SOLU-MEDROL STERILE POWDER 500MG/1000MG |
| SOLU-MEDROL STERILE POWDER 500MG/1000MG |
| URBASON SOLUBILE FORTE 1000 FOR INJ. |
| URBASON SOLUBILE FORTE 1000 FOR INJ. |
| URBASON (R) SOUBILE FORTE 1000 FOR INJECTION |
| URBASON (R) SOUBILE FORTE 1000 FOR INJECTION |
| HEMICORT 125MG |
| HEMICORT 2G |
| HEMICORT 40MG |
| SOLU-MEDROL STERILE POWDER 40MG |
| SOLU-MEDROL STERILE POWDER 125MG |
| STERILE SOLU-MEDROL |
| PREDNISOLONE TABLETS 5 MG S.S.P. |
| PELONINE CAPSULES "JOHNSON" |
| PREDONINE TABLETS |
| PREDOSON INJECTION 2.5% "Y.Y." |
| PREDOSON INJECTION 2.5% "Y.Y." |
| POLENIN TABLETS |
| SPALIN OINTMENT |
| PRESONON INJECTION "CHI SHENG" |
| PRESONON INJECTION "CHI SHENG" |
| PELONINE-R TABLETS "JOHNSON" |
| PALOSON TABLETS |
| PUKANLIN-S TABLETS |
| PELONINE TABLETS "JOHNSON" |
| DONISON TABLETS |
| DONISON TABLETS(鋁箔/膠箔) |
| PRENISONE TABLETS |
| PRENISONE POWDER |
| LYO-DONISON INJECTION |
| DONISON INJECTION |
| PROLILONE S.C. TABLETS |
| POKE SOLON TABLETS |
| SOLU-PRELON INJECTION |
| PRESOLONE TABLETS |
| PREDNISOLONE TABLETS |
| PREDNISOLONE 5MG TABLETS |
| PYRESON TABLETS |
| PYRESON TABLETS(瓶裝/84粒/瓶) |
| PREDNISOLONE TABLETS |
| PRENIN TABLETS "H.L." |
| PRESURIN TABLETS "S.C." |
| PREDNISOLONE TABLETS 5MG |
| PRELON TABLETS |
| PREDNISOLONE TABLETS "NYSCO" |
| PREDNISOLONE TABLETS |
| PREDNISOLONE INJECTION "N.K." |
| PREDNISOLONE INJECTION "N.K." |
| DOCAN TABLETS |
| SIULON INJECTION |
| PREDRON 5MG TABLETS "PRINCE" |
| PREDRON INJECTION "PRINCE" |
| PREDRON INJECTION "PRINCE" |
| PREDRON INJECTION "PRINCE" |
| PREDNISOLONE INJECTION "T.F." |
| PREDNISOLON TABLETS "ORIENTAL" |
| PREDNISOLONE TABLETS "SINTONG" |
| PREDNISOLONE TABLETS "SINTONG" (84粒/瓶) |
| PREKILINEN TABLETS |
| PRESONE TABLET "GOLDEN HORSE" |
| COLON TABLETS "D.T.S" |
| PREDNISOLONE TABLETS "CHINTENG" |
| PREDNISOLONE TABLETS "CHINTENG"(鋁箔/膠箔) |
| PREDNISOLONE TABLETS "WASHINGTON" |
| PREDNISOLONE TABLETS "N.Y." |
| KINGCORT TABLETS |
| PREDNISOLONE "KUANG NAN" |
| PRESON TABLETS "N.W." |
| TONFONRIN TABLETS "SWISS" |
| PREDNIAN TABLETS |
| KOSUTON INJECTION "SINTON" |
| PREDNISOLONE TABLETS "CHUNG MEI" |
| PREDONINE TABLETS |
| PREDONINE TABLETS(鋁箔/膠箔) |
| PELONINE CAPSULES "JOHNSON" |
| PREDONINE TABLETS |
| PREDONINE TABLETS(鋁箔/膠箔) |
| PELONINE-R TABLETS "JOHNSON" |
| PELONINE TABLETS "JOHNSON" |
| DONISON TABLETS |
| DONISON TABLETS(鋁箔/膠箔) |
| LYO-DONISON INJECTION |
| PYRESON TABLETS"F.Y." |
| PRESURIN TABLETS "S.C." |
| PREDNISOLONE INJECTION "T.F." |
| PREDNISOLONE TABLETS "SINTONG" |
| PREDNISOLONE TABLETS "CHINTENG" |
| PREDNISOLONE TABLETS "CHINTENG"(鋁箔/膠箔) |
| PREDNISOLONE TABLETS "WASHINGTON" |
| TONFONRIN TABLETS "SWISS" |
| PREDNISOLONE TABLETS "CHUNG MEI" |
| LEDERSPAN SUSPEND INJECTION 20MG/ML |
| ORICORT-IM INJECTION "ORIENTAL" |
| ORICORT-IM INJECTION "ORIENTAL" |
| ORICORT-IM INJECTION "ORIENTAL" |
| ORICORT-IM INJECTION |
| ORICORT INJECTION "ORIENTAL" |
| SUSWUN TABLETS |
| TRACO SUSPENSION 1% "CHI SHENG" |
| TRACON "CHI SHENG" |
| STACORT-A INJECTION 10MG "STANDARD" |
| STACORT-A INJECTION 10MG "STANDARD" |
| STACORT-A INJECTION 10MG "STANDARD" |
| TRIAMCINOLONE TABLETS "JEN SHENG" |
| AMCICORT TABLETS "N.W." |
| CHINACAN TABLETS |
| TOAMCILON TABLETS "S.S.P." |
| LARKCORT INJECTION 40MG/ML "LITA" |
| LARKCORT INJECTION 40MG/ML "LITA" |
| ASTERLONE INJECTION 10MG "ASTAR" |
| ASTARLONE INJECTION 40MG "ASTAR" |
| WINACORT-A TABLETS "WINSTON" |
| WINACORT-A INJECTION "WINSTON" |
| WINACORT-A INJECTION "WINSTON" |
| KONACOTO TABLETS "Y.C." |
| LARKCORT INJECTION 10MG/ML |
| LARKCORT INJECTION 10MG/ML |
| LARKCORT INJECTION 10MG/ML |
| STACORT-A INJECTION 40MG "STANDARD" |
| STACORT-A INJECTION 40MG "STSNDARD" |
| STACORT-A INJECTION 40MG "STANDARD" |
| NEWNACORT INJECTION |
| SINICORT IMM. INJECTION |
| SINICORT IM INJECTION |
| YUAKOLI TABLETS 4MG (TRIAMCINOLONE)"C.T." |
| LONACORT INJECTION 10MG/ML "C.Y." |
| OGECORT SUSPENDED INJECTIONS "Y.Y." |
| OGECORT SUSPENDED INJECTIONS "Y.Y." |
| SUZUNAN INJECTIONS "CHIU PO" |
| GRECORT-A INJECTION "G.L." |
| SINCORT SUSPENDED INJECTION |
| SINCORT SUSPENDED INJECTION |
| SINCORT SUSPENDED INJECTION |
| KERCORT TABLETS "CHIN TENG" |
| KERCORT TABLETS "CHIN TENG"(鋁箔/膠箔） |
| SHINCORT I.M. INJECTION "YUNG SHIN" |
| SHINCORT I.M. INJECTION "YUNG SHIN" |
| SHINCORT I.M. INJECTION "YUNG SHIN" |
| SHINCORT INJECTION (10MG/ML) |
| SHINCORT INJECTION "YUNG SHIN" |
| SHINCORT INJECTION "YUNG SHIN" |
| STERILE SHINOLON SUSPENDED INJECTION |
| STERILE SHINOLON SUSPENDED INJECTION |
| NEWNACORT INJECTION 40MG/ML |
| NEWNACORT INJECTION 40MG/ML |
| NICEFON TABLETS |
| KENARICIN TABLETS |
| SIVKORT SUSPENSION |
| SIVKORT SUSPENSION |
| BERLICORT SUSPENSION IM. INJECTION 40MG "PRINCE" |
| BERLICORT SUSPENSION IM. INJECTION 40MG "PRINCE" |
| BERLICORT SUSPENSION IM. INJECTION 40MG "PRINCE" |
| TRIAMCINOLONE SUSPENDED INJECTION 10MG "PRINCE" |
| TRIAMCINOLONE SUSPENDED INJECTION 10MG "PRINCE" |
| TRIAMCINOLONE SUSPENDED INJECTION 10MG "TAI YU" |
| TRIAMCINOLONE SUSPENDED INJECTION 10MG "TAI YU" |
| TRIAMCINOLONE SUSPENDED INJECTION 10MG "TAI YU" |
| TRIAMCINOLONE TABLETS "PFOSHEN" |
| TRIAMCINOLONE TABLETS "PATRON" |
| TRIAMCINOLONE SUSPENDED INJECTION 4% "TAI YU" |
| TRIAMCINOLONE SUSPENDED INJECTION 4% "TAI YU" |
| TRIAMCINOLONE SUSPENDED INJECTION 4% "TAI YU" |
| TRIAMCINOLONE SUSPENDED INJECTION 4% "TAI YU" |
| TRIAMCINOLONE TABLETS 4MG "C.R." |
| TRIAMCINOLONE TABLETS 4 MG "F.Y." |
| TRIAMCINOLONE TABLETS 4MG "F.Y."(鋁箔/膠箔) |
| WACICORT TABLETS "WST" (TRIAMCINOLONE) |
| SIVKORT STERILE SUSPENSION 40MG (TRIAMCINOLONE) |
| SIVKORT STERILE SUSPENSION 40MG (TRIAMCINOLONE) |
| SIVKORT STERILE SUSPENSION 40MG (TRIAMCINOLONE) |
| SIVKORT STERILE SUSPENSION 40MG (TRIAMCINOLONE) |
| LITONIN TABLETS (TRIAMCINOLONE) |
| TRINON TABLETS (TRIAMCINOLONE) "VEIMIN" |
| ANSULIN SUSPENDED INJECTION 40MG "S.T." |
| ANSULIN SUSPENDED INJECTION 40MG "S.T." |
| KENACORT TABLETS (TRIAMCINOLONE)"Y.Y." |
| TRIAMCINOLONE TABLETS "MERDER" |
| TRIALON INJECTION 40MG "C.C.P." |
| TRIALON INJECTION 40MG "C.C.P." |
| TRIALON INJECTION 40MG/ML |
| PANBICORT INJECTION (TRIAMCINOLONE) "PANBIOTIC" |
| PANBICORT INJECTION (TRIAMCINOLONE) "PANBIOTIC" |
| PANBICORT INJECTION (TRIAMCINOLONE) "PANBIOTIC" |
| TRIAMCINOLONE TABLETS "N.C.P." |
| TRIAMCINOLONE TABLETS "S.Y." |
| ANSUCORT INJECTION 10MG/ML "S.T."(TRIAMCINOLONE) |
| ANSUCORT INJECTION 10MG/ML "S.T."(TRIAMCINOLONE) |
| LIKAN TABLETS (TRIAMCINOLONE) "S.M." |
| TRICORT TABLETS (TRIAMCINOLONE) "S.C." |
| TRIAMCORT TABLETS "K.B." (TRIAMCINOLONE) |
| TRIAMCINOLONE TABLETS "C.H." |
| METROSONE TABLETS (TRIAMCINOLONE) "HETRO" |
| SINICORT INJECTION 10MG/ML (TRIAMCINOLONE ACETONIDE) |
| SINICORT INJECTION 10MG/ML (TRIAMCINOLONE ACETONIDE) |
| WINACORT INJECTION 40MG/ML (TRIAMCINOLONE ACETONIDE) "WINSTON" |
| WINACORT INJECTION 40MG/ML (TRIAMCINOLONE ACETONIDE) "WINSTON" |
| TRILONE TABLETS 4MG "H.H." (TRIAMCINOLONE) |
| TRILONE TABLETS 4MG "H.H." (TRIAMCINOLONE)(鋁箔/膠箔) |
| CORTICORD TABLETS 4MG (TRIAMCINOLONE) "T.F." |
| TRIAMCINOLONE TABLETS 4MG (TRIAMCINOLONE)"Y.C." |
| FULBIYEN TABLETS 4MG (TRIAMICINOLONE)"M.S." |
| TUNNACORT INJECTION 40MG/ML"T.F" |
| TUNNACORT INJECTION 40MG/ML "T.F." |
| TUNNACORT INJECTION 40MG/ML (TRIAMCINOLONE)"T.F." |
| KENACORT-A INTRAMUSCULAR INJECTION 40MG/ML (TRIAMCINOLONE ACETONIDE) |
| KENACORT-A INTRA-ARTICULAR-INTRADERMAL (TRIAMCINOLONE ACETONIDE) |
| KENACORT-A INTRA-ARTICULAR-INTRADERMAL (TRIAMCINOLONE ACETONIDE) |
| KENACORT-A INTRA-ARTICULAR-INTRADERMAL (TRIAMCINOLONE ACETONIDE) |
| KENACORT TABLETS 4MG (TRIAMCINOLONE) |
| KAMALON TAB 8MG |
| KENCORT TABLETS 4MG "EVEREST"(TRIAMCINOLONE) |
| TRIAMCINOLONE TABLETS 4MG "KOJAR" |
| HEALING BUCCAL TABLET 0.025MG"SHITEH"(TRIAMICINOLONE ACETONIDE) |
| SUZUNAN INJECTIONS 40MG/ML |
| TECORO TABLETS 0.025MG "EVEREST" |
| "TRIAMCINOLONE SUSPENDED INJECTION 10MG ""TAI YU""" |
| "TRIAMCINOLONE SUSPENDED INJECTION 10MG ""TAI YU""" |
| "TRIAMCINOLONE SUSPENDED INJECTION 10MG ""TAI YU""" |
| "TRIAMCINOLONE SUSPENDED INJECTION 4% ""TAI YU""" |
| "TRIAMCINOLONE SUSPENDED INJECTION 4% ""TAI YU""" |
| "TRIAMCINOLONE SUSPENDED INJECTION 4% ""TAI YU""" |
| "WINACORT-A TABLETS ""WINSTON""" |
| STACORT-A INJECTION 40MG "STANDARD" |
| STACORT-A INJECTION 40MG "STSNDARD" |
| KERCORT TABLETS "CHIN TENG" |
| KERCORT TABLETS "CHIN TENG"(鋁箔/膠箔） |
| SHINCORT I.M. INJECTION "YUNG SHIN" |
| SHINCORT I.M. INJECTION "YUNG SHIN" |
| SHINCORT INJECTION "YUNG SHIN" |
| SHINCORT INJECTION "YUNG SHIN" |
| NICEFON TABLETS |
| SIVKORT SUSPENSION |
| SIVKORT SUSPENSION |
| "TRIAMCINOLONE SUSPENDED INJECTION 10MG ""TAI YU""" |
| "TRIAMCINOLONE SUSPENDED INJECTION 10MG ""TAI YU""" |
| "TRIAMCINOLONE SUSPENDED INJECTION 10MG ""TAI YU""" |
| "TRIAMCINOLONE SUSPENDED INJECTION 4% ""TAI YU""" |
| "TRIAMCINOLONE SUSPENDED INJECTION 4% ""TAI YU""" |
| "TRIAMCINOLONE SUSPENDED INJECTION 4% ""TAI YU""" |
| TRIAMCINOLONE TABLETS 4MG "C.R." |
| TRIAMCINOLONE TABLETS 4 MG "F.Y." |
| SIVKORT STERILE SUSPENSION 40MG (TRIAMCINOLONE) |
| SIVKORT STERILE SUSPENSION 40MG (TRIAMCINOLONE) |
| SIVKORT STERILE SUSPENSION 40MG (TRIAMCINOLONE) |
| LITONIN TABLETS (TRIAMCINOLONE) |
| TRILONE TABLETS 4MG "H.H." (TRIAMCINOLONE) |
| TRILONE TABLETS 4MG "H.H." (TRIAMCINOLONE)(鋁箔/膠箔) |
| CORTICORD TABLETS 4MG (TRIAMCINOLONE) "T.F." |
| TRIAMCINOLONE TABLETS 4MG (TRIAMCINOLONE)"Y.C." |
| TUNNACORT INJECTION 40MG/ML "T.F." |
| KENCORT TABLETS 4MG "EVEREST"(TRIAMCINOLONE) |
| TRIAMCINOLONE TABLETS 4MG "KOJAR" |
| TECORO TABLETS 0.025MG "EVEREST" |
| KENACORT-A INTRAARTICULAR-INTRADERMAL |
| AFTACH |
| LEDERCORT TABLETS 4MG |
| LEDERCORT TABLETS 2MG |
| ARISTOSPAN PARENTERAL 20MG/ML |
| AFTACH |
| CORTCIN TABLETS "JOHNSON" |
| LEDERCORT TABLETS 2MG |
| LEDERCORT PARENTERAL INTRALESIONAL 25MG/ML |
| LEDERCORT TABLETS 4MG |
| PARAMESONE TABLETS 6MG |
| PARAMESONE TABLETS 2.0MG |
| PARAMESONE TABLETS 1.5MG |
| PARAME S.C. TABLETS |
| DISON TABLETS "VEI MIN" |
| DEXASONLIN INJECTION "SINTON" |
| DEXASONLIN INJECTION "SINTON" |
| DEXASONLIN INJECTION "SINTON" |
| DECANS TABLETS "C.M." |
| DECADOLONE TABLETS |
| MEDESONE TABLETS |
| DECAMIN INJECTION "ORIENTAL" |
| DEXAMETHASONE TABLETS "Y.S." |
| DECALON TABLETS "YU SHENG" |
| ERCAZON TABLETS |
| METHASONE INJECTION |
| DESON TABLETS "H.C." |
| DEXARON INJECTION 1MG |
| DEXARON INJECTION 1MG |
| DEXARON INJECTION 2MG |
| DEXARON INJECTION 2MG |
| DEXAMETHASONE INJECTION |
| DEXSONE TABLETS 0.5MG |
| TEANLANG TABLETS |
| DECOLIN TABLETS |
| DEXAMETHASONE TABLETS "ASTAR" |
| DECAMIN INJECTION 4MG/ML "ORIENTAL" |
| DECAMIN INJECTION 4MG/ML "ORIENTAL" |
| DECARON TABLETS |
| DEKESU TABLETS 0.5MG (DEXAMETHASONE)"F.S." |
| RONPILINE TABLETS "DAU CHI" |
| DEXAMETHASONE-21 INJECTION 1MG/ML"C.Y." |
| UCALON TABLETS 0.5MG "CHEN TA" (DEXAMETHASONE) |
| DECARONE TABLETS |
| DEXASON INJECTION 2MG/ML "LITA" |
| DEXASON INJECTION 4MG/ML "LITA" |
| DEXAZON TABLETS 0.5MG "GREAT ASIA" |
| DEXAMETHASONE TABLETS "MEIDER" |
| SUNDRON TABLETS |
| DEXAN TABLETS "H.S." |
| DECAN TABLETS "YUNG SHIN" |
| DECAN TABLETS "YUNG SHIN"(鋁箔) |
| MEXATON INJECTION 0.4% "Y.A." |
| DECAN INJECTION "YUNG SHIN" |
| DECAN INJECTION "YUNG SHIN" |
| DEXAMETHASONE TABLETS "S.C." |
| DICA TABLETS |
| DEXAMETHASONE INJECTION 4MG/ML "N.K." |
| DEXAMETHASONE INJECTION 4MG/ML "N.K." |
| SULININ TABLETS "K.N." |
| CAROLIN TABLETS "C.R." |
| DECARON S.C. TABLET 0.75MG (DEXAMETHASONE) "CHEN TA" |
| DEXON TABLETS "MAY SEE" |
| DEXAMETHASONE SYRUP "KODAK" |
| DEXAMETHASONE SYRUP "KODAK" |
| DEXAMETHASONE INJECTION "PANBIOTIC" |
| DEXAMETHASONE INJECTION "PANBIOTIC" |
| DEXAMETHASONE TABLETS "YUNG CHI" |
| DEXAMETHASONE TABLETS "WINSTON" |
| DEXAMETHASONE INJECTION "TAI YU" |
| DEXAMETHASONE INJECTION "TAI YU" |
| DEXAMETHASONE TABLETS "C.I." |
| DEXAMETHASONE INJECTION "WINSTON" |
| DECANON TABLETS 500 MCG "CHI SHENG" |
| DEXAMETHASONE TABLETS "PANBIOTIC" |
| DEXAMETHASONE TABLETS "S.Y." |
| DEXAMETHASONE INJECTION 4MG "TAI YU" |
| DEXAMETHASONE INJECTION 4MG "TAI YU" |
| DEXAMETHASONE TABLETS 4MG "STANDARD" |
| DEXAMETHASONE TABLETS "T.A." |
| DEXAMETHASONE INJECTIONS 4MG "ASTAR" |
| DEXAMETHASONE INJECTIONS 4MG "ASTAR" |
| DECALON TABLETS (DEXAMETH-ASONE) "NAN YA" |
| DEXAMETHASONE TABLETS "Y.C." |
| DEXAMETHASONE INJECTION 2MG/ML "N.K." |
| DEXAMETHASONE INJECTION 2MG/ML "N.K." |
| MEXASONE TABLETS "SHIN FONG" (DEXAMETHASONE) |
| DEXAMETHASONE INJECTION 4MG/ML "GENTLE" |
| DEXAMETHASONE INJECTION 4 MG/ML "GENTLE" |
| DEXAMETHASONE INJECTION 0.5% |
| DEXAMETHASONE INJECTION "Y.Y." |
| DEXAMETHASONE INJECTION "Y.Y." |
| DECONE TABLETS "SINPHAR"(DEXAMETHASONE) |
| DECONE TABLETS "SINPHAR"(DEXAMETHASONE)(鋁箔/膠箔) |
| DORISON TABLETS (DEXAMETHASONE) "ROYAL" |
| DORISON TABLETS (DEXAMETHASONE) "ROYAL"(鋁箔/膠箔) |
| DECONE INJECTION 5MG (DEXAMETHASONE) "SINPHAR" |
| DECALIN TABLETS (DEXAMETHASONE)"C.H." |
| DEXAMETHASONE TABLETS "KODAK" |
| DEXAMETHASONE INJECTION 0.4% "SWISS" |
| DEXAMETHASONE TABLETS "M.T." |
| DEXAMETHASONE PHOSPHATE INJECTION 5MG " K.S." |
| DEXAMETHASONE PHOSPHATE INJECTION 5MG " K.S." |
| HI-METHASONE INJECTION "TAH-AN" |
| DEXAMETHASONE TABLETS |
| DEXAMETHASONE TABLETS "TAI YU" |
| DECARON INJECTION (DEXAMETHASONE SODIUM PHOSPHATE) "Y.F." |
| DECARON INJECTION (DEXAMETHASONE SODIUM PHOSPHATE) "Y.F." |
| MID-METHASONE INJECTION "TAH-AN" |
| LICOSON INJECTION (DEXAMETHASONE SODIUM)"SINTON" |
| DEXAMETHASONE INJECTION 1MG/ML "TAI YU" |
| DEXAMETHASONE INJECTION 1MG/ML "TAI YU" |
| DEXAMETHASONE INJECTION 1MG/ML "TAI YU" |
| DEXAMETHASONE TABLETS 0.5MG "KODAK" |
| DEXAMETHASONE TABLETS 0.5MG (DEXAMETHASONE) "L.S." |
| TEANLANG TABLETS 0.75MG |
| DEXAMETHASONE INJECTION 4MG "Y.Y." |
| DEXAMETHASONE INJECTION 4MG "Y.Y." |
| DECA TABLETS 0.75MG (DEXAMETHASONE) "S.T." |
| DEXAMETHASONE TABLETS |
| DEXAMETHASONE INJECTION 2MG/ML "Y.A" |
| METHASONE INJECTION 5MG/ML (DEXAMETHASONE PHOSPHATE) "VPP" |
| METHASONE TABLETS 0.25MG (DEXAMETHASONE) "VPP |
| DEXADROL TABLETS (DEXAMETHASONE)"T.F." |
| DEXAMETHASONE S.C. TABLETS 0.5MG "M.T." |
| DEXAMETHASONE S.C. TABLETS 0.5MG "M.T." （鋁箔/膠箔） |
| DEXAMETHASONE TABLETS "M.T." |
| UNISONE INJ. 4MG/ML (DEXAMETHASONE) "UNION" |
| UNISONE INJECTION 4MG/ML (DEXAMETHASONE) "UNION" |
| UNISONE INJECTION 4MG/ML (DEXAMETHASONE) "UNION" |
| DEXONE TABLETS 0.5MG (DEXAMETHASONE) "CENTER" |
| SHUAYAN TABLETS 0.5MG (DEXAMETHASONE)"Y.C." |
| NUGACHENIN TABLETS 0.5MG "C.T." (DEXAMETHASONE) |
| DECA TABLETS 0.75MG "KOJAR" (DEXAMETHASONE) |
| DECA TABLETS 0.75MG (DEXAMETHASONE)"KOJAR"(鋁箔/膠箔) |
| DECA TABLETS 0.5MG "KOJAR" (DEXAMETHASONE) |
| DECA TABLETS 0.5MG (DEXAMETHASONE) "KOJAR"(鋁箔/膠箔) |
| DECADLIN TABLETS 0.5MG (DEXAMETHASONE) "M.S" |
| DECORON TABLETS 0.5MG "EVEREST" (DEXAMETHASONE) |
| DEXAROID INJECTION 24MG/ML (DEXAMETHASONE) |
| DECORON F.C. TABLET 0.75MG "EVEREST" (DEXAMETHASONE) |
| DEXARON INJECTION 5MG/ML 'S.Y.' |
| DEXONE TABLETS 0.5MG (DEXAMETHASONE) "CENTER" |
| DECADLIN TABLETS 0.5MG (DEXAMETHASONE) "KOJAR" |
| NEOSONE TABLETS 0.5MG |
| ANCOM TABLETS "D.T.S." |
| DECANS TABLETS "C.M." |
| DEXAMETHASONE TABLETS "Y.S." |
| DECALON TABLETS "YU SHENG" |
| DESON TABLETS "H.C." |
| DEXARON INJECTION 2MG |
| DEXARON INJECTION 2MG |
| DEXAMETHASONE INJECTION |
| "DEXAMETHASONE TABLETS ""ASTAR""" |
| DEXASON INJECTION 4MG/ML "LITA" |
| DEXAMETHASONE TABLETS "MEIDER" |
| DEXAN TABLETS "H.S." |
| DECAN TABLETS "YUNG SHIN" |
| DECAN TABLETS "YUNG SHIN"(鋁箔/膠箔) |
| DECAN INJECTION "YUNG SHIN" |
| DEXAMETHASONE INJECTION 4MG/ML "N.K." |
| DEXAMETHASONE INJECTION 4MG/ML "N.K." |
| DEXAMETHASONE INJECTION 4MG/ML "N.K." |
| CAROLIN TABLETS "C.R." |
| DEXAMETHASONE INJECTION "PANBIOTIC" |
| DEXAMETHASONE INJECTION "PANBIOTIC" |
| "DEXAMETHASONE TABLETS ""WINSTON""" |
| "DEXAMETHASONE INJECTION ""TAI YU""" |
| "DEXAMETHASONE INJECTION ""TAI YU""" |
| "DEXAMETHASONE TABLETS ""S.Y.""" |
| "DEXAMETHASONE INJECTION 4MG ""TAI YU""" |
| "DEXAMETHASONE INJECTION 4MG ""TAI YU""" |
| DEXAMETHASONE TABLETS 4MG "STANDARD" |
| DEXAMETHASONE INJECTION 4MG/ML "GENTLE" |
| DEXAMETHASONE INJECTION 4 MG/ML "GENTLE" |
| DEXAMETHASONE INJECTION 4 MG/ML "GENTLE" |
| DEXAMETHASONE INJECTION 0.5% |
| DEXAMETHASONE INJECTION "Y.Y." |
| DEXAMETHASONE INJECTION "Y.Y." |
| DEXAMETHASONE INJECTION "Y.Y." |
| DECONE TABLETS "SINPHAR"(DEXAMETHASONE) |
| DECONE TABLETS "SINPHAR"(DEXAMETHASONE)(鋁箔/膠箔) |
| "DORISON TABLETS (DEXAMETHASONE) ""ROYAL""" |
| "DORISON TABLETS (DEXAMETHASONE) ""ROYAL""(鋁箔/膠箔)" |
| DECALIN TABLETS (DEXAMETHASONE)"C.H." |
| "DEXAMETHASONE TABLETS ""M.T.""" |
| "DEXAMETHASONE TABLETS ""TAI YU""" |
| "DEXAMETHASONE INJECTION 1MG/ML ""TAI YU""" |
| "DEXAMETHASONE INJECTION 1MG/ML ""TAI YU""" |
| "DEXAMETHASONE INJECTION 1MG/ML ""TAI YU""" |
| TEANLANG TABLETS 0.75MG |
| DEXAMETHASONE INJECTION 4MG "Y.Y." |
| DEXAMETHASONE INJECTION 4MG/ML "Y.Y." |
| DECA TABLETS 0.75MG (DEXAMETHASONE) "S.T." |
| METHASONE INJECTION 5MG/ML (DEXAMETHASONE PHOSPHATE) "VPP" |
| DEXADROL TABLETS (DEXAMETHASONE)"T.F." |
| DEXAMETHASONE S.C. TABLETS 0.5MG "M.T." |
| "DEXAMETHASONE TABLETS ""M.T.""" |
| SHUAYAN TABLETS 0.5MG (DEXAMETHASONE)"Y.C." |
| DECA TABLETS 0.75MG "KOJAR" (DEXAMETHASONE) |
| DECA TABLETS 0.75MG (DEXAMETHASONE)"KOJAR"(鋁箔/膠箔) |
| DECA TABLETS 0.5MG "KOJAR" (DEXAMETHASONE) |
| DECA TABLETS 0.5MG (DEXAMETHASONE) "KOJAR"(鋁箔/膠箔) |
| DECORON TABLETS 0.5MG "EVEREST" (DEXAMETHASONE) |
| DECORON F.C. TABLET 0.75MG "EVEREST" (DEXAMETHASONE) |
| DEXAMETHASONE INJECTION 0.5% "CYH" |
| ANCOM TABLETS "D.T.S." |
| DECOLONE TABLETS |
| LIMESON TABLETS "Y.C." |
| LIMESON TABLETS "Y.C."(鋁箔/膠箔) |
| SELFTISON INJECTION |
| DEFLAME |
| ORADEXON INJECTION |
| ORADEXON INJECTION |
| DEXAMETHASONE ACET. AQUOSUSP INJECTION |
| ORADEXON TABLETS |
| DEXACORTIN INJECTION |
| DEXAMETHASONE INJECTION "ASTRADEX" |
| DECADRON PHOSPHATE INJECTION |
| DECADRON TABLET 0.5MG |
| SOFRADEX DROPS |
| DEXAMETHASNE SODIUM PHOSPHATE 4MG/ML 5ML |
| DEXAMETHASNE SODIUM PHOSPHATE 4MG/ML 10ML |
| DEXAMETHASNE SODIUM PHOSPHATE 4MG/ML 30ML |
| SOFRADEX DROPS |
| DEXAMETHASONE TABLETS 0.5MG "STANDARD" |
| DEXAZONE TABLETS "JOHNSON" |
| DICOSHEUN TABLETS |
| DECASON TABLETS |
| DEXADON-21-P INJECTION 4MG/ML "PRINCE" |
| DEXAMETHASONE TABLETS "C.C.P." |
| DEXAMETHASONE TABLETS "C.C.P."(鋁箔/膠箔) |
| DEXAMETHASONE INJECTION "H.S.C." |
| DEXAMETHASONE INJECTION "H.S.C." |
| DEXAMETHASONE TABLETS |
| DEMESON TABLETS 0.5MG "F.Y." |
| DEMESON TABLETS 0.5MG "F.Y."(瓶裝/42粒/瓶)、(瓶裝/84粒/瓶) |
| DECOTONG TABLETS |
| DECOTONG INJECTION |
| DECOTON TABLETS "H.L." |
| DEXAMETHASONE TABLETS " Y.Y." |
| DEXAROID INJECTION 4 MG "CHI SHENG" |
| DEXAROID INJECTION 4 MG "CHI SHENG" |
| DEXAMESONE INJECTION 4MG "CHI SHENG." |
| DEXAROID INJECTION 1 MG "CHI SHENG" |
| DEXAROID INJECTION 1 MG "CHI SHENG" |
| DEXAROID INJ. 1MG/ML |
| ROCOLONE TABLETS "S.C." |
| DEXASON TABLETS 0.5MG "LITA" |
| DEXAMETHASONE 1MG/ML INJECTION "N.K." |
| DEXAMETHASONE 1MG/ML INJECTION "N.K." |
| DEXASOLEN TABLETS |
| NEOSOLON TABLETS |
| DEXAMESONE INBJECTION 10MG |
| DEXAMESONE INJECTION 4MG "T.F." |
| DEXAMESONE INJECTION 4MG/ML "T.F." |
| DEXARON TABLETS |
| DEXASONE TABLETS |
| DEXASOLON TABLETS |
| DEXAMETHASON TABLETS |
| DEXMESONE TABLETS "KINGDOM" |
| DEXAMETHASONE-21-P INJECTION "ASTAR" |
| DEXAMETHASONE-21-P INJECTION "ASTAR" |
| DEXARON INJECTION |
| DEXARON INJECTION |
| DEXAMETHASONE TABLETS "SHITEH" |
| DEXADON -21.9 INJECTION "PRINCE" |
| DEXADON TABLETS "PRINCE" |
| DEXADON-P INJECTION 1MG/ML "PRINCE" |
| DEXAMETHASONE TABLETS "JEN SHENG" |
| DEXASONE INJECTION "SINTON" |
| DEXASONE INJECTION "SINTON" |
| DEXASONE INJECTION "SINTON" |
| DEXAMETHASONE TABLETS "SENTAI" |
| DECASONE TABLETS "GOLDEN HORSE" |
| DEXAMETHASONE "SWISS" |
| ANCOM TABLETS"D.T.S" |
| DEXASONE TABLETS "SINTON" |
| KOVIN TONG TABLETS |
| KOVIN TONG TABLETS(鋁箔) |
| CANALON TABLETS |
| DEXAMETHASONE TABLETS 0.5MG "STANDARD" |
| DEXAMETHASONE TABLETS 0.5MG "STANDARD" |
| DEXAZONE TABLETS "JOHNSON" |
| DEXAMETHASONE TABLETS "C.C.P." |
| DEXAMETHASONE TABLETS "C.C.P."(鋁箔/膠箔) |
| DEMESON TABLETS 0.5MG "F.Y." |
| DEXAMETHASONE TABLETS " Y.Y." |
| DEXASON TABLETS 0.5MG "LITA" |
| DEXAMESONE INJECTION 4MG "T.F." |
| DEXAMESONE INJECTION 4MG/ML "T.F." |
| DEXARON INJECTION |
| DEXARON INJECTION |
| DEXAMETHASONE "SWISS" |
| KOVIN TONG TABLETS |
| KOVIN TONG TABLETS (鋁箔/膠箔) |
| VANMESON TABLETS |
| PANMESON INJECTION |
| PANMESON INJECTION |
| BETAMETHASONE TABLETS "LITA" |
| BETA-DEXALONE TABLETS |
| DELTONIN INJECTION "N.K." |
| DELTONIN INJECTION "N.K." |
| BETAMETHASONE TABLETS |
| LONGCORT TABLETS |
| LONGCORT INJECTION |
| BETASHIN TABLETS |
| BETAMETHASONE TABLETS |
| BETASONE TABLETS |
| BETASON TABLETS "M.T." |
| DOUBLE BETASON INJECTION "LITA" |
| DOUBLE BETASON INJECTION "LITA" |
| BEINSON TABLETS "YUNG CHI" |
| RINMESON TABLETS |
| BETAMETHASONE TABLETS "Y.C." |
| BETAMETHASONE TABLETS "YU SHENG" |
| BETAMETHASONE INJECTION |
| METASONE TABLETS "H.H." |
| METASONE TABLETS "H.S."(鋁箔/膠箔) |
| BETAMETHASONE SODIUM PHOSPHATE INJECTION "PRINCE" |
| BETAMETHASONE SODIUM PHOSPHATE INJECTION "PRINCE" |
| SUMIN TABLETS 0.5MG (BETAMETHASONE) "F.S." |
| BETAMETHASONE TABLETS "PRINCE" |
| VETHASONE INJECTION "S.Y." |
| VETHASONE INJECTION "S.Y." |
| BETAMETHASONE TABLETS "NCP" |
| BETASONE INJECTION "K.S." |
| BETASONE TABLETS "SHIN FONG" |
| BETAMETHASONE TABLETS "M.T." |
| BETASON TABLETS 0.5MG "CHEN TA" (BETAMETHASONE) |
| UPTHASONE TABLETS "JINUP" |
| BETAMETHASONE TABLETS "JOHNSON" |
| VETHASONE SUSPENDED INJECTION "S.Y." |
| BETAMETHASONE TABLETS "C.M." |
| BETAMETHASONE TABLETS "SHINLON" |
| BETAMETHASONE TABLETS "YUNG SHIN" |
| BETAMETHASONE TABLETS "C.I." |
| BETAMETHASONE TABLETS "UNIVERSAL" |
| BETASONE TABLETS (BETAMETHASONE) "GCPC" |
| BETAMETHASONE TABLETS "HONTEN" |
| ORIDERON INJECTION "ORIENTAL" |
| BETASONE INJECTION (DI-BETAMETHASONE) |
| BETASONE INJECTION (DI-BETAMETHASONE) |
| BETALIN TABLETS (BETAMETHASONE) |
| BETAMETHASONE TABLETS "T.A." |
| BETAMETHASONE TABLETS 0.5MG "C.T." |
| HEMODIN SUPPOSITORIES "PATRON" |
| BETAMETHASONE SUSPENDED INJECTION "GENTLE" |
| BETAMETHASONE TABLETS "ORIENTAL" |
| BETAMETHASONE INJECTION "Y.Y." |
| BETAMETHASONE INJECTION "Y.Y." |
| BETAMETHASONE TABLETS "S.Y." |
| BETAMETHASONE INJECTION "TAI YU" |
| BETAMETHASONE INJECTION "TAI YU" |
| BETAMETHASONE INJECTION "TAI YU" |
| METHASONE TABLETS (BETAMETHASONE) "H.C." |
| BETAMETHASONE TABLETS "ASTAR" |
| CHIMIN TABLETS 0.5MG "Y.C." (BETAMETHASONE) |
| BETAMETHASONE INJECTION 4MG/ML "Y.A" |
| BETAMETHASONE INJECTION 4MG/ML "Y.A" |
| WINBETA TABLETS 0.5MG (BETAMETHASONE) "WINSTON" |
| BETASONE TABLETS 0.5MG (BETAMETHASONE)"Y.Y" |
| BETASON INJECTION 4MG/ML "LITA" (BETAMETHASONE) |
| BETASON INJECTION 4MG/ML "LITA" (BETAMETHASONE) |
| BETASONE TABLETS 0.6MG (BETAMETHASONE) "WEIDAR" |
| RINDERON SUSPENSION 5MG/ML |
| RINDERON SUSPENSION 5MG/ML |
| RINDERON SUSPENSION 5MG/ML 0 |
| RINDERON INJECTION 4MG/ML (BETAMETHASONE) |
| RINDERON INJECTION 4MG/ML (BETAMETHASONE) |
| RINDERON INJECTION 4MG/ML (BETAMETHASONE) |
| METASONE TABLETS 0.5MG (BETAMETHASONE) "N.W." |
| BETASONE TABLETS 0.5MG (BETAMETHASONE) "CENTER" |
| BUFENCON INJECTION |
| BUFENCON INJECTION |
| BECASONE TABLETS 0.6MG "KOJAR" (BETAMETHASONE) |
| BATONSU INJECTION 4MG/ML (BETAMETHASONE) "SINTON" |
| LIFUMIN CAPSULES 0.5MG "L.D." (BETAMETHASONE) |
| BEDASON TABLETS "S.C" |
| RINDERON TABLETS |
| NORHEUM TABLETS "ROOT" |
| BETAMETHASONE TABLETS "LITA" |
| BETA-DEXALONE TABLETS |
| DELTONIN INJECTION "N.K." |
| DELTONIN INJECTION "N.K." |
| BETAMETHASONE TABLETS "YU SHENG" |
| METASONE TABLETS "H.S." |
| "VETHASONE INJECTION ""S.Y.""" |
| "BETAMETHASONE TABLETS ""M.T.""" |
| BETAMETHASONE TABLETS "JOHNSON" |
| BETAMETHASONE TABLETS "YUNG SHIN" |
| BETAMETHASONE TABLETS "YUNG SHIN" (84粒/瓶) |
| BETALIN TABLETS (BETAMETHASONE) |
| "BETAMETHASONE TABLETS ""S.Y.""" |
| "BETAMETHASONE INJECTION ""TAI YU""" |
| "BETAMETHASONE INJECTION ""TAI YU""" |
| "BETAMETHASONE INJECTION ""TAI YU""" |
| "BETAMETHASONE TABLETS ""ASTAR""" |
| CHIMIN TABLETS 0.5MG "Y.C." (BETAMETHASONE) |
| BETASON INJECTION 4MG/ML "LITA" (BETAMETHASONE) |
| BETASON INJECTION 4MG/ML "LITA" (BETAMETHASONE) |
| BETASONE TABLETS 0.6MG (BETAMETHASONE) "WEIDAR" |
| RINDERON INJECTION 4MG/ML (BETAMETHASONE) |
| BUFENCON INJECTION |
| BUFENCON INJECTION |
| BECASONE TABLETS 0.6MG "KOJAR" (BETAMETHASONE) |
| BECASONE TABLETS 0.6MG (BETAMETHASONE) "KOJAR"(鋁箔/膠箔) |
| RINDARON TABLETS 0.5MG"EVEREST" (BETAMETHASONE) |
| BEDASON TABLETS "S.C" |
| RINDERON TABLETS |
| "NORHEUM TABLETS ""ROOT""" |
| DIPROSPAN INJECTION |
| DIPROSPAN INJECTION |
