# Supplementary material for: Incidence and risk factors for recurrent Henoch-Schönlein purpura in children from a 16-year nationwide database
Source: Pediatr Rheumatol Online J. 2018 Apr 16;16:25. doi: 10.1186/s12969-018-0247-8 (PMC5902957; doi:10.1186/s12969-018-0247-8)
Supplement: Supplementary file 2 — Table S2. The percentage and duration of steroid use in each episode. (DOCX 18 kb) [file 12969_2018_247_MOESM2_ESM.docx]

**Table S2. The percentage and duration of steroid use in each episode**

|  | Total case numbers | Numbers and percentage of  steroid usage n (%) | Average duration of  steroid usage (days) | *P* value*^¶^* |
| --- | --- | --- | --- | --- |
|  |  |  |  | < 0.001 |
| Only 1 episode | 838 | 342 (40.8%) | 6.9 |  |
| At least 2 episodes | 77 | 43 (55.8%) | 10.1 |  |
| At least 3 episodes | 27 | 20 (74.1%) | 16.3 |  |
| More than 3 episodes | 60 | 41 (68.3%) | 23.1 |  |

*^¶^*Kruskal-Wallis Test
